# Prediction of Complications and Surgery Duration in Primary Total Hip Arthroplasty Using Machine Learning: The Necessity of Modified Algorithms and Specific Data

**DOI:** 10.3390/jcm11082147

**Published:** 2022-04-12

**Authors:** Igor Lazic, Florian Hinterwimmer, Severin Langer, Florian Pohlig, Christian Suren, Fritz Seidl, Daniel Rückert, Rainer Burgkart, Rüdiger von Eisenhart-Rothe

**Affiliations:** 1 Department of Orthopaedics and Sports Orthopaedics, Klinikum Rechts der Isar, Technical University of Munich, 80333 Munich, Germany; florian.hinterwimmer@tum.de (F.H.); severin.langer@mri.tum.de (S.L.); florian.pohig@mri.tum.de (F.P.); christian.suren@tum.de (C.S.); burgkart@tum.de (R.B.); eisenhart@tum.de (R.v.E.-R.); 2 Institute for AI and Informatics in Medicine, Technical University of Munich, 80333 Munich, Germany; daniel.rueckert@tum.de; 3 Department of Trauma Surgery, Klinikum Rechts der Isar, Technical University of Munich, 80333 Munich, Germany; friedrich.seidl@mri.tum.de

**Keywords:** artificial intelligence, machine learning, hip surgery, total hip arthroplasty, supervised learning

## Abstract

Background: Machine Learning (ML) in arthroplasty is becoming more popular, as it is perfectly suited for prediction models. However, results have been heterogeneous so far. We hypothesize that an accurate ML model for outcome prediction in THA must be able to compute arthroplasty-specific data. In this study, we evaluate a ML approach applying data from two German arthroplasty-specific registries to predict adverse outcomes after THA, after careful evaluations of ML algorithms, outcome and input variables by an interdisciplinary team of data scientists and surgeons. Methods: Data of 1217 cases of primary THA from a single center were derived from two German arthroplasty-specific registries between 2016 to 2019. The XGBoost algorithm was adjusted and applied. Accuracy, sensitivity, specificity and AUC were calculated. Results: For the prediction of complications, the ML algorithm achieved an accuracy of 80.3%, a sensitivity of 31.0%, a specificity of 89.4% and an AUC of 64.1%. For the prediction of surgery duration, the ML algorithm yielded an accuracy of 81.7%, a sensitivity of 58.2%, a specificity of 91.6% and an AUC of 89.1%. The feature importance indicated non-linear outcomes for age, height, weight and surgeon. No relevant linear correlations were found. Conclusion: The attunement of input and output data as well as the modifications of the ML algorithm permitted the development of a feasible ML model for the prediction of complications and surgery duration.

## 1. Introduction

Global trends indicate a rising prevalence of total hip arthroplasties (THA). Similarly, revision arthroplasties will increase, which are more expensive and are associated with poorer outcomes and more complications [1,2,3]. THA procedures in the United States are estimated to grow by 174% until the year 2030, while the number of THA revisions will double by 2026 [4].

These findings suggest that the identification of patients at risk for revision is crucial for the individual patient as well as for health care providers in regard to the expected unsustainable expenditures. Hence, risk stratification models have been developed to face this problem. However, current risk analysis tools in arthroplasty have been moderately accurate so far in predicting adverse outcomes [5]. A major problem with these existing risk stratification models is that they do not sufficiently include specific risk factors in arthroplasty, which together may significantly contribute to the failure of the arthroplasty. However, it is hard to quantify such a revision probability as multilinear correlations are difficult to realize in conventional risk stratification models. Therefore, more comprehensive and specific risk analysis tools are urgently needed.

Machine Learning (ML) evolved from learning theory and is capable of detecting multilinear correlations in complex datasets [6]. ML applications in arthroplasty are gaining popularity as they may potentially improve preoperative decision making [7]. 

Shah et al. investigated administrative data from 89,986 adults who underwent primary THA to predict major complications after primary THA by comparing different ML algorithms and logistic regression models [8]. Although the authors concluded superior discriminative ability of a ML approach, they concluded that the predictive performance was not sufficient for clinical applicability. They further indicated that the most important variables for their algorithm were malnutrition, dementia and cancer. The investigated administrative database, however, does not provide arthroplasty-specific variables. It is therefore obvious that no arthroplasty-specific variables were considered relevant by their algorithm. Hence, the relevance of malnutrition, dementia and cancer as the most relevant parameters for major complications after primary THA must be further discussed, especially in view of the clinical applicability of such prediction models. The prediction of clinically relevant outcomes such as the occurrence of revisions or complications is intricate as predicting seldom outcomes is not just limited by the amount, but also by the complexity of the applied data. Hence, the prediction of rare outcomes such as adverse events in arthroplasty might not be feasible using only administrative data. We therefore consider the implementation of parameters specific to arthroplasty to be crucial.

Our hypothesis is that an accurate prediction model must have a balanced calibration of specific input data, the algorithm and the outcome labels. We assume that a close collaboration between data scientists and orthopedic surgeons is crucial in this context as extensive knowledge of data science methodology and arthroplasty procedures is necessary. Hence, in this study, we evaluate a ML approach applying data from two German arthroplasty-specific registries to predict adverse outcomes after THA after careful evaluations of ML algorithms, outcome and input variables by an interdisciplinary team of data scientists and surgeons.

## 2. Materials and Methods

### 2.1. Data Source

The German Society for Orthopaedics and Orthopaedic Surgery (Deutsche Gesellschaft für Orthopädie und Orthopädische Chirurgie) has established two national associations to improve the quality of care in arthroplasty. The German Arthroplasty Registry (Endoprothesenregister Deutschland (EPRD)) reports implant-related data of hip and knee replacements and EndoCert monitors compliance with structural, process and outcome quality standards in hospitals and certifies medical facilities accordingly. All patients undergoing primary THA between 2016 to 2019 at our EndoCert-certified institution and giving consent to participate in EPRD were included in this study (*n* = 1217).

### 2.2. Data Screening, Cleaning and Preparation

Before analysis, both datasets from EPRD and EndoCert were screened collaboratively by a data scientist (F.H.) and an orthopedic surgeon (I.L.) regarding their relevance and applicability for ML analysis. Twelve parameters were chosen to be relevant for the prediction of two outcome variables (complications and irregular surgery duration), which were classified by EndoCert (Table 1). In total, 6.1% (1053/17,038) of the data points were missing and could not be retrieved from the clinical information system. In total, 28.8% (1053/3651) of the data points regarding weight, height and therefore BMI are missing, because they were only added to the EPRD registry in 2017. The data samples were still kept for the final dataset, as a model which can cope with a limited number of missing data points was chosen.

### 2.3. Input Parameters

The complete dataset consists of 1217 patient cases from our hospital undergoing primary THA between 2016 to 2019. Input parameters and their sources are presented in Table 1.

### 2.4. Outcome

After detailed substantive and methodological evaluation, two outcome labels were classified as feasible on the basis of the input data: complications and irregular surgery duration. Complications were derived from the EndoCert dataset, consisting of the following postoperative events within 90 days after implantation: deviation of the mechanical axis ±3°, periprosthetic infection, dislocation, periprosthetic fracture, revision surgery, thromboembolism, neurologic complications and mortality. As the total of complications occurred in only 13.2% (161/1217) of cases, the outcome was translated in a binary classification. Similarly, for prediction of surgery duration, the binary classification according to EndoCert as regular or irregular (either <40 min or >100 min) was applied. An irregular duration occurred in 21.9% (267/1217) (Table 2). To increase the quality of the presented observational study and its prediction model, we reported in accordance with the Transparent Reporting of a Multivariable Prediction Model for Individual Prognosis or Diagnosis (TRIPOD) guidelines [9] and the Strengthening the Reporting of Observational Studies in Epidemiology (STROBE) statement [10]. 

### 2.5. Statistical Analysis

Linear correlations in the dataset were searched using Spearman’s rank-order correlation coefficient using Python 3.9.6. A |ρ| > 0.5 concludes a significant direct or indirect correlation between two parameters. 

### 2.6. Machine Learning Model

**Figure 1:**
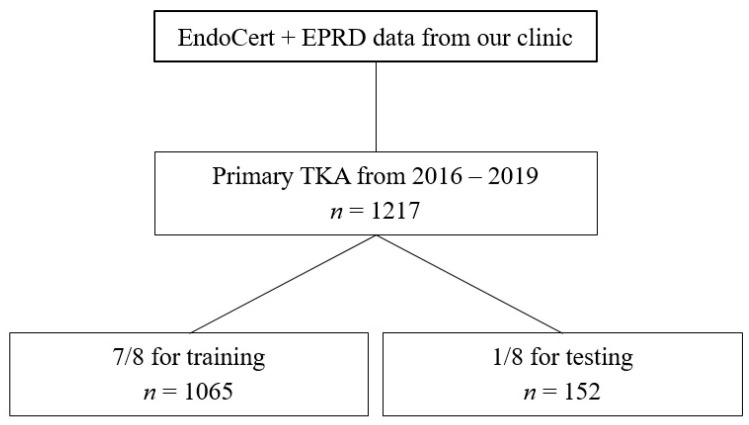
Flowchart describing training and testing datasets.

XGBoost [11,12], a state-of-the-art implementation of gradient boosting decision trees, was chosen for the task at hand. It is capable of high performance and managing missing data points. To obtain statistical significance, cross-validation was performed by splitting the data into a specified number of folds. At least one fold is used exclusively for testing, and the other folds are used for training. All folds must be disjointed to avoid cross-contamination. After several runs, each data sample was used exactly once for testing. Then, the results were averaged to obtain more realistic and stable metric values. For this study, a hyperparameter search for the optimal number of folds (data split) was performed, resulting in a split of eight folds (one for testing, seven for training) (Figure 1). To counteract the significant class imbalance, loss weighting was applied: the loss of the entity with fewer samples (in this study, the occurrence of complications/irregular surgery duration) was weighted higher than the dominant entity (no complications/regular surgery duration). A feature importance was calculated to support a deeper understanding of the algorithm’s predictions and to retrieve insight into data patterns. Feature importance refers to a technique that assigns a relative score to input parameters based on how useful they are in predicting a target.

Implementation of the code for the ML model as well as for the statistical analysis was realized with Python 3.9.6 (https://python.org, accessed on 6 April 2022) and the Scikit-learn library (https://scikit-learn.org, accessed on 6 April 2022). The source code for this study is provided on GitHub (https://github.com/FlorianH3000/ML_tabdata, accessed on 10 April 2020).

## 3. Results

Overall, 1217 patients with a median age of 67 (13, 97) were included in this study. In total, 672 (55.2%) patients were female and 545 (44.8%) were male. The median BMI was at 23 with a standard deviation of 11.7. Indications for THA were classified as primary osteoarthritis (817, 67.1%), dysplasia (129, 10.6%), fracture (16, 1.3%), femoral head necrosis (80, 6.6%), posttraumatic osteoarthritis (46, 3.8%) and tumor/metastasis (129, 10.6%). The surgeries were performed by 17 different main surgeons with a share of 0.1% up to 26.0% of all surgeries. The experience level of surgeons specified by the EndoCert initiative was distributed from 1.4% (level 1), 9.1% (level 2), 0.1% (level 3), 16.4% (level 4) to 73.0% (level 5). In total, 225 complications in 161 cases occurred (41 cases with multiple complications): inclination > 50° (66/29.3%), infection (41/18.2%), dislocation (8/3.6%), fracture of greater trochanter (23/10.2%), periprosthetic fracture (22/9.8%), revision surgery 40/17.8%, thromboembolism (10/4.4%), mortality (8/3.6%) and neurologic complications (7/3.1%). Surgery duration lasted from 38 to 339 min. The distribution of the outcome labels is summarized in Table 2.

**Figure 2:**
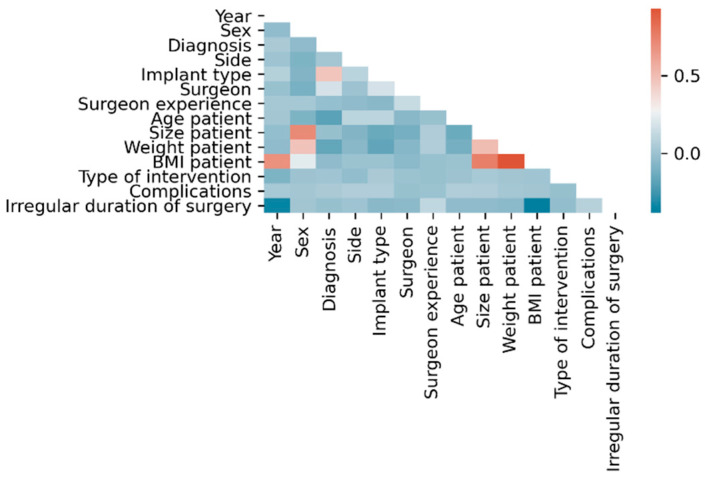
Correlation matrix of input parameters.

Within the dataset, the following linear correlations were found using classical statistical methods considering |ρ| > 0.5 as statistically relevant: BMI with each year (ρ = 0.68), height (ρ = 0.75) and weight (ρ = 0.93), height with sex (ρ = −0.71) and weight with height (ρ = 0.51) (Figure 2).

**Figure 3:**
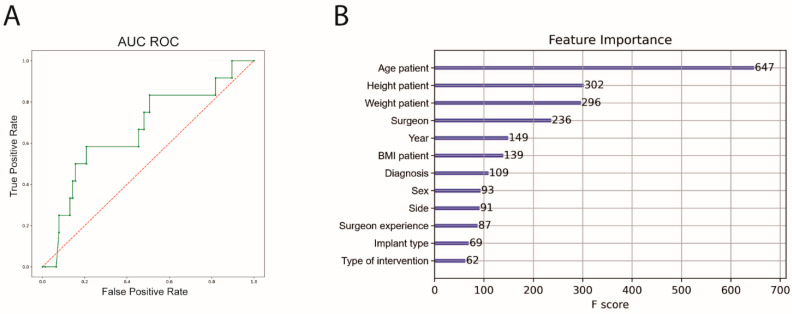
AUC ROC and feature importance for complication prediction. (**A**) Machine Learning algorithm for complication prediction (green), AUC ROC = 0.64, (**B**) feature importance of the prediction model; AUC ROC = area under the curve receiver operating characteristics. BMI, body mass index.

For ML, cross-validation with an 8-fold split was performed (training *n* = 1065, test *n* = 152). The performance of the ML algorithm for predicting complications showed a sensitivity of 31.0%, a specificity of 89.4%, an accuracy of 80.3% and an area under the receiver operating curve (AUC ROC) of 64.1%. Feature importance was subsequently calculated. The highest feature importance for predicting complications was patient age, height and weight before surgeon (Figure 3).

**Figure 4:**
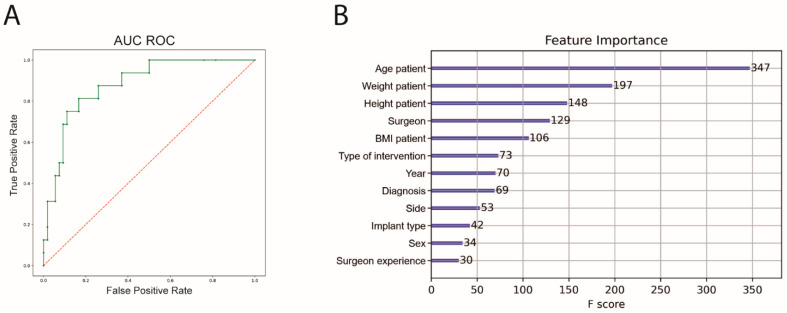
AUC ROC and feature importance for prediction of irregular surgery duration. (**A**) Machine Learning algorithm for irregular surgery prediction (green), AUC ROC = 0.89, (**B**) feature importance of the prediction model; AUC ROC = area under the curve receiver operating characteristics. BMI, body mass index.

An 8-fold cross-validation was also performed for the prediction of irregular surgery duration. The performance of the ML algorithm showed a sensitivity of 58.2%, a specificity of 91.6%, an accuracy of 81.7% and an AUC ROC of 89.1%. Patient age, weight and height before intervention had the highest significance for predicting complications (Figure 4). The outcome metrics for both prediction models are summarized in Table 3.

## 4. Discussion

Current risk stratification tools do not provide actionable intelligence in clinical practice as their results cannot be directly transferred to clinical cases. Therefore, ML approaches capable of improving preoperative decision making are gaining popularity. However, no clinically applicable models have been developed yet, as predictive performances were heterogeneously reported [8,13,14]. The most important finding of this study is that a feasible ML model was developed for the prediction of complications and irregular surgical durations in primary THA with a high accuracy by using data from two arthroplasty registries. In this context, we highlight our novel methodological approach: input parameters, algorithm and output variables were carefully balanced by an orthopedic surgeon and a data scientist. The most immediate consequence for everyday clinical practice is that a data scientist should be involved in clinical processes.

ML models have predictive power, meaning they can correctly predict outcomes over the course of time as they respond and adapt to complex data inputs. The aspect of learning is achieved by function optimization: the algorithm seeks to determine the best parameter constellations that will minimize the error when confronted with a novel dataset. This training process relies on sufficient data that are complex enough to reveal specific parameter constellations. In data science, the lack of data complexity may be countered by large data volumes. Hence, most of ML applications in orthopedics utilize imaging data [6]. Conversely, data concerning the outcomes of THA are stored in tabular form which, however, has a significantly lower information density than imaging data, even compared to extensive administrative data available in registries and hospital information systems.

Hence, the outcome to be predicted and the ML algorithm should be suited to the applied dataset. Current tabular data volumes concerning THA forbid more complex applications such as deep learning. Hence, even if arthroplasty-specific data are considered, it does not necessarily imply that the prediction of multifactorial events such as revision must be feasible. We assume that simpler surrogates are easier to predict with less extensive datasets. Furthermore, the selection of a ML algorithm has a significant influence on the model performance [15]. A variety of algorithms for specific tasks exists and the choice of a suitable algorithm is difficult. 

Noteworthy, a high number of different input parameters do not necessarily improve the performance of a ML algorithm. Quite the contrary, feature selection techniques exist to avoid overfitting and improve model performance by restricting the amount of input parameters (i.e., cross-validation, data augmentation, L1/L2 regularization or removal of layers) [16]. From these methodological considerations, we hypothesized that a ML application intended to predict specific outcomes based on tabular data in orthopedics requires well-considered, specific input data. Hence, we assume that the methodological and substantial discussion regarding the outcomes a priori to the computation of the ML algorithm is mandatory in order to obtain meaningful results that seem clinically and methodologically plausible. For this reason, complications in total were chosen as the outcome label, since in our dataset single complications as listed by EndoCert occurred too rarely. Hence, a representative prediction of specific complications was not technically feasible. Similarly, the low data density for irregular surgery durations forbids the definition of a cut-off value applying continuous variables. Hence, a binary classification into regular and irregular durations was established, with which accurate results could finally be derived.

Interestingly, the prediction of complications yielded worse AUC and sensitivity compared to the duration prediction. This is most likely due to the fact that irregular durations were found almost twice as often as complications (13.2% vs. 21.9%). Hence, a greater class imbalance of cases with and without complications was present so that a highly sensitive prediction in the limited test set of only 152 cases was not achieved.

A particular challenge in the application of ML is that the decisions made by the algorithm are not intelligible in retrospect. Such algorithms are referred to as black box models, whose predications are based on a combination of variables from complex functions which cannot be reproduced from neither the code nor the results. Black box models have high predictive power, but if black box models are superior to interpretable models is currently the subject of scientific research in the field of data science [17]. In this context, the feature importance indicates to which extent a variable has been weighted by the ML model without indicating a causal relationship nor unbiased associations. An interpretation of the results and an evaluation of significant correlations have to be performed subsequently. In this study, the highest feature importance for both complication and duration prediction was age, height and weight. Younger patient age and obesity have previously been described as risk factors in primary THA [18,19,20]. However, a significant finding of this study is that these results were derived from the ML algorithm and have not been found by classic statistical analysis using a logistic function model in this dataset, indicating the successful feasibility of this approach. 

Interestingly, the particular surgeon showed the fourth highest feature importance in both prediction models. It is reasonable to assume that the individual surgeon has an impact on the success of the procedure. However, it has been difficult to incorporate a surrogate for the individual expertise of a surgeon into more conventional risk stratification models so far. One of the strengths of ML is its ability to compute such daily clinical data. However, even if the results are clinically comprehensible, they must be analyzed with a data scientist for confounding and systemic errors. In this study, out of 17 surgeons, 3 surgeons performed 58% of the operations. Ten surgeons performed less than 10% and eight surgeons performed less than 2% of the operations, respectively, resulting in a relevant class imbalance. Complications occurring in cases of the latter surgeons are weighted far greater and therefore most likely contribute to the high feature importance. However, whether this result means that an experienced surgeon contributes significantly to fewer complications and regular surgery times or that the presence of various inexperienced surgeons biases this result by raising the feature importance, cannot be finally assessed as the inherent decisions of the ML algorithm cannot be retrieved. In this context, it is interesting to note that the experience of the surgeon as classified by EndoCert has only a poor feature importance in this study. However, this finding has clinical relevance as the surgeon and his experience should be included in further clinical evaluations of risk factors in THA. Hence, these results are reason to (1) further investigate this correlation with conventional statistical models and (2) to tackle the problem of class imbalance in ML applications in arthroplasty. The latter may be assessed by external validation of the algorithm with larger datasets. However, it is highly unlikely that class imbalance will be fully resolved in medicine, since the occurrence of adverse events will naturally always be more seldom than the number of successful treatments. This is a desirable scenario from a statistical point of view, since it corresponds to a Gaussian distribution and does not induce a bias to the dataset. From a data science perspective, however, a class imbalance impedes an increase in the results and has to be tackled through larger datasets so that the class imbalance becomes less impactful and the algorithm can still learn patterns from the data at hand. We therefore conclude that a profound discussion of the dataset and the derived outcomes is critical, especially regarding the outlook of possible ML applications as decision support systems in orthopedics, where decisions are at high stakes and the data basis is rather small from a data science perspective. 

Interestingly, diagnosis and implant type had low feature importance, although we assumed otherwise. Whether substantive or methodological causes account for the missing impact of these parameters in both ML algorithms remains unclear due to the black box model. The most common diagnosis was primary osteoarthritis with 67.1% and the most common implant type was primary implants with 76.6%. A potential impact of these parameters for the outcome prediction has to be revaluated with either a more balanced dataset or by a detailed analysis of the various diagnoses and implant types in separate datasets.

This study has several limitations. The first and utmost relevant limitation is the low data volume from a data science perspective. The data preparation as described above resulted in an aggregation of data in categorical variables. This results in the loss of potentially relevant information through the simplification of the dataset. Elaborate information such as implant components differentiated by a manufacturer could not be adequately addressed by this approach because there simply were not enough separate datasets available. The generalization of these data may have led to a selection bias. Second, the availability of data in both arthroplasty-specific registries was restricted. Weight and height were only available in the registry since 2017. As both parameters demonstrated relevant results in this study, we did not exclude them from the analysis despite the missing data points. For this reason, however, we chose a ML algorithm capable of handling missing data points. Although no relevant deviations are expected in the missing weight and height data, the results may theoretically differ after inclusion. Third, the results of this study are not generalizable as the algorithm is not externally validated. However, we applied several statistical measures (e.g., cross validation, data split) to provide significance. In this context, we highlight that we aimed to conduct a feasibility study with single-center data. The results must therefore be interpreted under these circumstances.

In conclusion, after thorough calibration of input and output data as well as the definition of outcome labels by an orthopedic surgeon and a data scientist, we were able to build an accurate ML model for the prediction of complications and irregular surgery durations for primary THA. Age, height, weight and the performing surgeon showed the highest feature importance for both complication and duration prediction. These parameters, however, were not assessed by conventional statistical evaluations. Therefore, we recommend assessing arthroplasty-specific data in future clinical practice to build an in-depth database for the clinical application of ML prediction models. For the successful implementation of these data in ML applications, a data scientist should be directly involved in the clinical workflows. Interdisciplinary analysis by a data scientist and an orthopedic surgeon to comprehend the significance of identified parameters outside the scope of the presented ML model is crucial to allow for accurate prediction models.

## Figures and Tables

**Table 1:** Description of input parameters.

*Parameters*			*Source*
***Nominal Data, n (%)***			
***Sex***			EndoCert/EPRD
Male	545	44.8%	
Female	672	55.2%	
**Diagnosis**			EndoCert/EPRD
Primary osteoarthritis	817	67.1%	
Dysplasia	129	10.6%	
Femoral neck fracture	16	1.3%	
Femoral head necrosis	80	6.6%	
Posttraumatic osteoarthritis	46	3.8%	
Tumour/metastasis	129	10.6%	
**Side**			EndoCert/EPRD
Left	590	48.5%	
Right	627	51.5%	
**Implant type**			EPRD
Primary implant	932	76.6%	
Revision implant	231	19.0%	
Tumour implant	54	4.4%	
**Surgeon**			EndoCert
Surgeon 1	20	1.6%	
Surgeon 2	1	0.1%	
Surgeon 3	92	7.6%	
Surgeon 4	317	26.0%	
Surgeon 5	6	0.5%	
Surgeon 6	1	0.1%	
Surgeon 7	62	5.1%	
Surgeon 8	43	3.5%	
Surgeon 9	1	0.1%	
Surgeon 10	2	0.2%	
Surgeon 11	10	0.8%	
Surgeon 12	8	0.7%	
Surgeon 13	96	7.9%	
Surgeon 14	216	17.7%	
Surgeon 15	182	15.0%	
Surgeon 16	89	7.3%	
Surgeon 17	71	5.8%	
**Ordinal Data, *n* (%)**			
**Experience Level of Surgeon**			EndoCert
1 Resident	17	1.4%	
2 Fellow	111	9.1%	
3 Attending-Junior	1	0.1%	
4 Attending—Main Surgeon *	199	16.4%	
5 Attending—Senior Surgeon *	889	73.0%	
**Year**			EndoCert/EPRD
2016	324	26.6%	
2017	394	32.4%	
2018	282	23.2%	
2019	217	17.8%	
**Ratio Data, median (standard deviation)**			
Age	67	13.8	EndoCert/EPRD
Height (in cm)	170	88.0	EPRD
Weight (in kg)	80	17.4	EPRD
BMI	23	11.7	EPRD

* “Attending—Main Surgeon” corresponds to “Hauptoperateur” (>50 operation per year) and “Attending—Senior Surgeon” corresponds to “Senior-Hauptoperateur” (>100 operation per year) as defined by Endocert; Body Mass Index (BMI), Endoprothesenregister Deutschland (EPRD).

**Table 2:** Distribution of outcome labels.

*Outcome Label, n (%)*			*Source*
Complications	161	(13.2%)	EndoCert
Irregular duration of surgery	267	(21.9%)	EndoCert

**Table 3:** Outcome metrics for the prediction models of complications and irregular surgery duration.

Prediction of	Accuracy	Sensitivity	Specificity	AUC *
Complications	80.3	31.0	89.4	64.1
Irregular Duration	81.7	58.2	91.6	89.1

* AUC = area under the receiver operating curve.

## Data Availability

The source code for this study is provided on GitHub (https://github.com/FlorianH3000/ML_tabdata, accessed on 10 April 2020).

## References

[1] Maradit Kremers H., Larson D.R., Crowson C.S., Kremers W.K., Washington R.E., Steiner C.A., Jiranek W.A., Berry D.J. (2015). Prevalence of Total Hip and Knee Replacement in the United States. J. Bone Jt. Surg. Am. Vol..

[2] Bozic K.J., Ong K., Kurtz S., Lau E., Vail T.P., Rubash H., Berry D. (2016). Short-term Risk of Revision THA in the Medicare Population Has Not Improved with Time. Clin. Orthop. Relat. Res..

[3] Vanhegan I.S., Malik A.K., Jayakumar P., Islam S.U., Haddad F.S. (2012). A financial analysis of revision hip arthroplasty: The economic burden in relation to the national tariff. J. Bone Jt. Surg. Br. Vol..

[4] Kurtz S., Ong K., Lau E., Mowat F., Halpern M. (2007). Projections of Primary and Revision Hip and Knee Arthroplasty in the United States from 2005 to 2030. J. Bone Jt. Surg. Am..

[5] Manning D.W., Edelstein A.I., Alvi H.M. (2016). Risk Prediction Tools for Hip and Knee Arthroplasty. J. Am. Acad. Orthop. Surg..

[6] Cabitza F., Locoro A., Banfi G. (2018). Machine Learning in Orthopedics: A Literature Review. Front. Bioeng. Biotechnol..

[7] Hinterwimmer F., Lazic I., Suren C., Hirschmann M.T., Pohlig F., Rueckert D., Burgkart R., von Eisenhart-Rothe R. (2022). Machine learning in knee arthroplasty: Specific data are key—A systematic review. Knee Surg. Sports Traumatol. Arthrosc..

[8] Shah A.A., Devana S.K., Lee C., Kianian R., van der Schaar M., SooHoo N.F. (2021). Development of a Novel, Potentially Universal Machine Learning Algorithm for Prediction of Complications After Total Hip Arthroplasty. J. Arthroplast..

[9] Klionsky D.J., Abdel-Aziz A.K., Abdelfatah S., Abdellatif M., Abdoli A., Abel S., Abeliovich H., Abildgaard M.H., Abudu Y.P., Acevedo-Arozena A. (2021). Guidelines for the use and interpretation of assays for monitoring autophagy (4th edition). Autophagy.

[10] Vandenbroucke J.P., von Elm E., Altman D.G., Gøtzsche P.C., Mulrow C.D., Pocock S.J., Poole C., Schlesselman J.J., Egger M. (2007). Strengthening the Reporting of Observational Studies in Epidemiology (STROBE): Explanation and Elaboration. Epidemiology.

[11] Bentéjac C., Csörgo A., Martínez-Muñoz G. (2019). A Comparative Analysis of XGBoost. arXiv.

[12] Chen T., Guestrin C. XGBoost: A Scalable Tree Boosting System. Proceedings of the 22nd ACM SIGKDD International Conference on Knowledge Discovery and Data Mining.

[13] Kunze K.N., Polce E.M., Sadauskas A.J., Levine B.R. (2020). Development of Machine Learning Algorithms to Predict Patient Dissatisfaction After Primary Total Knee Arthroplasty. J. Arthroplast..

[14] Thornton C., Hutter F., Hoos H.H., Leyton-Brown K. Auto-WEKA: Combined selection and hyperparameter optimization of classification algorithms. Proceedings of the 19th ACM SIGKDD International Conference on Knowledge Discovery and Data Mining.

[15] Saeys Y., Inza I., Larrañaga P. (2007). A review of feature selection techniques in bioinformatics. Bioinformatics.

[16] Rudin C., Radin J. (2019). Why Are We Using Black Box Models in AI When We Don’t Need To? A Lesson from An Explainable AI Competition. Harv. Data Sci. Rev..

[17] Corbett K.L., Losina E., Nti A.A., Prokopetz J.J.Z., Katz J.N. (2010). Population-Based Rates of Revision of Primary Total Hip Arthroplasty: A Systematic Review. PLoS ONE.

[18] Haynes J., Nam D., Barrack R.L. (2017). Obesity in total hip arthroplasty. Bone Jt. J..

[19] Liu W., Wahafu T., Cheng M., Cheng T., Zhang Y., Zhang X. (2015). The influence of obesity on primary total hip arthroplasty outcomes: A meta-analysis of prospective cohort studies. Orthop. Traumatol. Surg. Res..

[20] Prokopetz J.J.Z., Losina E., Bliss R.L., Wright J., Baron J.A., Katz J.N. (2012). Risk factors for revision of primary total hip arthroplasty: A systematic review. BMC Musculoskelet. Disord..

